# Photolysis of a Caged, Fast-Equilibrating Glutamate Receptor Antagonist, MNI-Caged *γ*-D-Glutamyl-Glycine, to Investigate Transmitter Dynamics and Receptor Properties at Glutamatergic Synapses

**DOI:** 10.3389/fncel.2018.00465

**Published:** 2018-12-05

**Authors:** Francisco Palma-Cerda, George Papageorgiou, Boris Barbour, Céline Auger, David Ogden

**Affiliations:** ^1^Brain Physiology Lab, UMR8118 Université Paris Descartes, Paris, France; ^2^The Francis Crick Institute, London, United Kingdom; ^3^Institut de Biologie de l’Ecole Normale Supérieure (IBENS), Ecole Normale Supérieure, CNRS, INSERM, PSL University, Paris, France

**Keywords:** photolysis, synaptic transmission, caged fast antagonist, glutamate receptor, climbing fiber, Purkinje cell, cerebellum, nitroindoline

## Abstract

Fast uncaging of low affinity competitive receptor antagonists can in principle measure the timing and concentration dependence of transmitter action at receptors during synaptic transmission. Here, we describe the development, synthesis and characterization of MNI-caged *γ*-D-glutamyl-glycine (*γ*-DGG), which combines the fast photolysis and hydrolytic stability of nitroindoline cages with the well-characterized fast-equilibrating competitive glutamate receptor antagonist *γ*-DGG. At climbing fiber-Purkinje cell (CF-PC) synapses MNI-caged-γ-DGG was applied at concentrations up to 5 mM without affecting CF-PC transmission, permitting release of up to 1.5 mM γ-DGG in 1 ms in wide-field flashlamp photolysis. In steady-state conditions, photoreleased γ-DGG at 0.55–1.7 mM inhibited the CF first and second paired EPSCs by on average 30% and 60%, respectively, similar to reported values for bath applied γ-DGG. Photolysis of the L-isomer MNI-caged *γ*-L-glutamyl-glycine was ineffective. The time-course of receptor activation by synaptically released glutamate was investigated by timed photolysis of MNI-caged-γ-DGG at defined intervals following CF stimulation in the second EPSCs. Photorelease of γ-DGG prior to the stimulus and up to 3 ms after showed strong inhibition similar to steady-state inhibition; in contrast γ-DGG applied by a flash at 3–4 ms post-stimulus produced weaker and variable block, suggesting transmitter-receptor interaction occurs mainly in this time window. The data also show a small and lasting component of inhibition when γ-DGG was released at 4–7 ms post stimulus, near the peak of the CF-PC EPSC, or at 10–11 ms. This indicates that competition for binding and activation of AMPA receptors occurs also during the late phase of the EPSC, due to either delayed transmitter release or persistence of glutamate in the synaptic region. The results presented here first show that MNI-caged-γ-DGG has properties suitable for use as a synaptic probe at high concentration and that its photolysis can resolve timing and extent of transmitter activation of receptors in glutamatergic transmission.

## Introduction

The time scale and spatial distribution of transmitter release are important in determining both the speed and independence of information transfer between pre- and postsynaptic elements in synaptic transmission. Synapses with a single or few release sites and short-lived transmitter transients show greater independence and postsynaptic specificity than multisite synapses that activate a larger postsynaptic volume. This distinction has been formalized as synaptic vs. parasynaptic (“spillover” or volume transmission; Szapiro and Barbour, 2007, 2009; Coddington et al., 2014) and relates directly to the time for which transmitter interacts with postsynaptic receptors following release. Fast, timed application of an antagonist to the post-synaptic receptors would enable dissection of the time-course of receptor activation to distinguish modes of transmission. However, there are few examples of caged photolabile derivatives of receptor antagonists applied in neuroscience, although they are potentially useful tools in probing synaptic function on the time scale of transmission. They offer the possibility of timed release and competition with transmitter binding at postsynaptic receptors, thereby giving time-resolved data of transmitter concentration at the target receptors. With further development of selective ligands there is the possibility of targeting specific signaling pathways as has been done with selective agonists (Palma-Cerda et al., 2012).

To be useful caged antagonists require fast photochemistry relative to the synaptic timescale, with uncaging rates >10,000 s^−1^ following a light pulse. Additionally, to facilitate repeated applications in wide-field photolysis, it is also desirable that the photoreleased antagonist dissociates rapidly from receptors, requiring a low affinity, contrary to the high affinity generally useful for pharmacological studies at synapses. Because of the dependence of block on transmitter concentration, the low affinity competitive receptor antagonists also permit a measure of the relative concentration of the competing neurotransmitter during transmission. Here, we report the development and application of a fast-equilibrating caged glutamate receptor antagonist to study excitatory synaptic mechanisms. Photolysis is with the well-established nitroindoline photochemistry, which is both fast (half-time for release 0.15 μs; Morrison et al., 2002) and stable to hydrolysis (Papageorgiou et al., 1999). Nitroindoline photochemistry has been applied to synaptic physiology since 1999 and is well established as an effective experimental tool in neuroscience.

Previous use of the low-affinity antagonist *γ*-D-glutamyl-glycine (γ-DGG, Davies and Watkins, 1981) has been in the analysis of L-glutamate concentration and time-course at synapses. In one study, Liu et al. (1999) applied a concentration-ratio analysis to show low affinity competitive antagonism by γ-DGG with L-glutamate at hippocampal AMPA-R synapses (estimated equilibrium dissociation constant *K* = 0.55 mM) and used the dependence of block on glutamate concentration to show fast equilibration of γ-DGG with receptors. At climbing fiber-Purkinje cell (CF-PC) synapses Wadiche and Jahr (2001) used the glutamate concentration dependence of γ-DGG block to test multivesicular release during paired responses. The analysis was based on fast application patch data and kinetic simulations, estimating a fast dissociation rate constant of 10^4^ s^−1^. The use of low affinity antagonists to assess neurotransmitter concentration and time-course (Clements et al., 1992) has been evaluated by Beato (2008) at glycinergic synapses and reviewed by Scimemi and Beato (2009). In simulations they showed a correlation between transmitter concentration and exposure time that prevents separate determination and requires additional information, such as the effects of transporter inhibition, to obtain independent parameter estimate. Furthermore, independent determination of the postsynaptic gating properties is required for analysis to estimate transmitter timing and concentration; these are usually obtained from patch experiments and do not take account of possible differences between excised patch and post-synaptic receptor properties, resulting for instance from different receptor subunit/auxiliary protein compositions. In this context the precise timing and quantitation of photorelease proposed here would be useful since data are gathered at the synapse of interest.

In this report, we describe the chemical synthesis and characterization of MNI-caged *γ*-DGG and tests for interference with synaptic transmission at high (mM) concentration at cerebellar climbing fiber to Purkinje cell synapses. Both enantiomers *γ-DGG* and *γ*-LGG were prepared in caged form. The latter endogenous dipeptide (Kanazawa et al., 1965) served as a readily available model reagent in the synthesis and has been reported to have a lower affinity for glutamate receptors (Francis et al., 1980; Davies and Watkins, 1981), making it useful as a control probe. Structures of the two caged compounds are shown in the Supplementary Information. The results show that MNI-caged γ-DGG can be used at concentrations up to 5 mM to release mM concentrations by flashlamp or laser photolysis and that timing of competition with synaptic L-glutamate can be resolved with 1 ms precision with flashlamp photolysis. This article describes the properties of MNI-caged γ-DGG in relation to synaptic investigations and presents results of transmitter timing and duration at the CF-PC synapse with wide-field flashlamp photolysis. Photolysis of MNI-caged amino acids is readily adapted to localized laser photolysis (Trigo et al., 2009) to yield faster time and better spatial resolution.

## Materials and Methods

### Ethical Approval

Sprague–Dawley rats were provided by Janvier (St Berthevin, France) and subsequently housed in agreement with the European Directive 2010/63/UE regarding the protection of animals used for experimental and other scientific purposes. Experimental procedures were approved by the French Ministry of Research and the ethical committee for animal experimentation of Paris Descartes.

#### Slice Preparation

Experiments were made with parasagittal slices 200 μm thick cut from the cerebellum of Sprague-Dawley rats, age 12–21 days. Slices were prepared and stored at 34°C throughout in a solution containing (mM): 115 NaCl, 2.5 KCl, 1.3 NaH_2_PO_4_, 26 NaHCO_3_, 0.1 L-ascorbic acid, 25 glucose, 1 MgSO_4_, 2 CaCl_2_ equilibrated with 95% O_2_–5% CO_2_.

#### Electrophysiological Recording

A Hepes-buffered saline was used containing a low concentration of bicarbonate to maintain intracellular pH, composition (mM): 130 NaCl, 4 KCl, 2.5 NaHCO_3_, 10 Hepes, 25 glucose, 0.1 ascorbic acid, 1 mM MgSO_4_ and 2 mM CaCl_2_, pH 7.3 with NaOH, perfused at 5 mL/min. Once a recording was established perfusion was stopped and the cage, dissolved at 20 mM in Hepes-buffered isotonic saline, added to the bath volume. Air was blown over the surface of the static bath solution to produce mixing of the caged compound; ambient pCO_2_ is equilibrated at 2.5 mM bicarbonate and pH is controlled by Hepes. The presence of bicarbonate facilitates intracellular pH regulation by CO_2_ diffusion across the membrane. The intracellular solution for Purkinje neurons was (in mM): 150 K gluconate, 10 Hepes, 0.01 EGTA, 2.5 MgCl_2_, 2 ATPNa_2_ and 0.4 GTPNa, 5 mM QX314, pH adjusted to 7.3 with KOH and osmolality to 300 mosmol/kg. Alexa 488 20 μM was included to record cell morphology (Andor Ixon EMCCD; Oxford, UK). Recordings were at 21–24°C. The preparation was viewed with a Zeiss Axioskop1FS (Oberkochen, Germany) with Olympus 40× 0.8w objective. To avoid photolysis, transmitted illumination was long pass filtered at 425 nm. The optical arrangement is described in Supplementary Figure S2.

Recording pipettes had resistances of 2.5–3.5 MΩ. Whole cell voltage clamp recordings from Purkinje neurons were with an Axopatch 200B amplifier (Molecular Devices, San Jose, CA, USA). Series resistance was 7–10 MΩ and compensated 60%–80%. The pipette potential was held at −60 to −30 mV, data were not corrected for the junction potential of 12 mV. Data acquisition was with a CED P1401 interface (Cambridge Electronic Design, Cambridge, UK) LP filtered at 3 kHz, sampled at 50 kHz with WINWCP (Dr. John Dempster, University of Strathclyde) and analyzed in Igor Pro 7 (Wavemetrics, Lake Oswego, OR, USA).

For climbing fiber (CF) stimulation, cathodal pulses of 50 μs duration and supramaximal amplitude were delivered from a constant voltage stimulator (DS2, Digitimer, Welwyn UK) through glass pipettes with 2 MΩ resistance filled with bath solution, positioned at the surface of the slice in the granule cell layer. Voltage thresholds were 30–70 V (15–35 μA) determined regularly during recordings. The stimulus comprised two pulses separated by 40 ms or 150 ms to test paired pulse depression. To inhibit Na-current in the Purkinje neuron with CF stimulation the intracellular solution contained 5 mM QX314.HCl salt. The extracellular solution was supplemented with 3 μM SR 95531 and 50 μM D-AP5 to block GABAA and NMDA receptors, respectively, or as specified in Figure legends.

### Flash Photolysis

**Figure 1:**
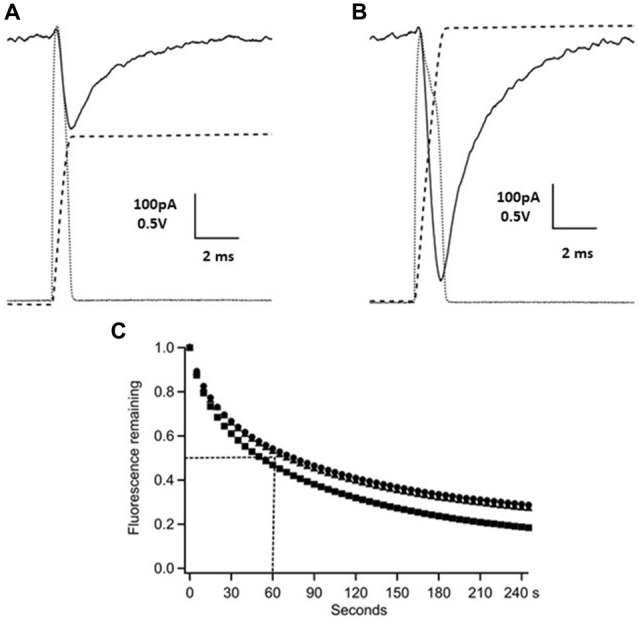
Time-course of *γ*-D-glutamyl-glycine (*γ*-DGG) concentration during and following photorelease. Panels **(A,B)** show photodiode records of the flash (dotted gray traces, scale bar 0.5 V) and their integrals over the pulse representing the time-course of the *γ-DGG* concentration (dashed traces), and the whole cell current generated by photolysis of MNI-caged *γ-DGG* in a Purkinje neuron at −60 mV (black traces, scale bar 100 pA). Panel **(A)** shows the response to a low intensity flash as used in synaptic experiments, producing 14% conversion to release *γ-DGG* and proton concentrations of 0.65 mM. Panel **(B)** shows the response to a strong flash releasing 1.3 mM *γ-DGG* and protons. The bath concentration of MNI-caged *γ-DGG* was 4.6 mM in **(A,B)**. **(C)** Following wide-field photolysis of NPE-HPTS the time-course of diffusional loss of HPTS fluorescence was monitored from a region defined by an aperture in a conjugate image plane corresponding to 10 μm in the slice. HPTS and amino acids have similar diffusion coefficients in extracellular space. A single flash released HPTS from 100 μM NPE-HPTS equilibrated in the slice. Fluorescence of photoreleased HPTS (em 525/50 nm) from the 10 μm spot was measured every 5 s thereafter, corrected for background counts and normalized to the maximum fluorescence immediately after the flash. The data are from three trials, the dotted lines show the mean half-time for the fluorescence decline, estimated as 60 s; the time-course is expected to be similar for photoreleased *γ-DGG*.

Flash photolysis was through the condenser of the transmitted light path with a Xenon Arc Flashlamp (Rapp Optoelectronic, Hamburg, Germany) producing a 0.5–1.5 ms light pulse, bandpass filtered 275–395 nm, energy measured as 120 mJ on discharge at 300V with three capacitors. Because of the high cage concentration it is not feasible to use epi-illumination for near UV wide spectrum photolysis, the light is absorbed too strongly by mM cage over 3 mm pathlength. The optical arrangement and calibration procedures are summarized in Supplementary Figure S2. The flashlamp was coupled with a 3 mm liquid light guide into the transmitted-illumination path by a silica lens and 425 nm LP dichroic reflector and focused by a silica condenser through the slice to illuminate uniformly a 200 μm diameter region at the top surface. Calibration of photolysis was in separate experiments by comparison with photolysis of caged fluorophore NPE-HPTS (the nitrophenylethyl ether of HPTS) which becomes fluorescent on uncaging to release free HPTS (exc 470/40 nm, em 525/50 nm; see Supplementary Figure S2 caption in Supplementary Information; also Canepari et al., 2001). Briefly, NPE-HPTS at 100 μM in borate buffer (10 mM pH 9) is suspended by stirring into Sylgard 184 resin to form vesicles of 5–20 μm diameter that cure to form a thin layer on coverslips. The progressive increase in fluorescence in each vesicle with consecutive flashlamp pulses depends on the efficiency of photolysis in the microscope at the flashlamp parameters used, the conversion/flash obtained by fitting an exponential under conditions where bleaching of released HPTS is minimal. The conversion of MNI-glutamate relative to NPE-HPTS with wide-field flashlamp illumination and band-pass near UV filter was determined as 2.1, and will be the same for MNI-caged γ-DGG or MNI-caged *γ*-LGG, since no additional chromophores are present. A further correction was applied for transmission of photolysis light through the slice, estimated from transmission of the flashlamp pulse as 32% in the molecular layer of cerebellar slices 200 μm thick from 18 day rats, 40% at 15 days and 44% at 13 days. At the end of the experiment the bath concentration of MNI-γ-DGG was measured in a microliter spectrophotometer (Nanodrop, ThermoFisher Scientific) from the absorbance at 322 nm with molar absorption coefficient 4800 M^−1^cm^−1^. To obtain the concentration released, the cage concentration was multiplied by the conversion/flash of NPE-HPTS at the flash intensity used, multiplied by the factor of 2.1 for relative efficiencies of MNI-glutamate to NPE-HPTS with this excitation spectrum, and by the transmission factor through the slice dependent on the age of the animal. Measurements of several vesicles in the microscope field showed that photolysis was uniform over the 200 μm field viewed by the camera (Andor Ixon, Oxford Instruments, Oxford, UK). Conversion efficiencies for MNI-caged-γ-DGG at the flashlamp settings used here, as in Figures 1A,B, were estimated as 14%/flash for 300 V one capacitor and 30%/flash for 300 V three capacitors in 13-day 200-μm thick slices.

### Drugs

Drugs used were D-AP5, SR 95531, NBQX, Cyclothiazide, Amiloride and QX314.HCl from Tocris Bioscience UK or Sigma-Aldrich.

### Chemical Synthesis and Photochemistry of MNI-Caged *γ*-D-Glutamylglycine

**SCHEME 1:**
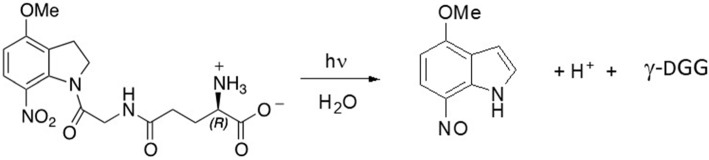
Photolysis of MNI-caged γ-DGG releases γ-DGG, a nitrosoindole by-product and a proton for each caged molecule photolysed.

Synthesis and purification of both D and L optical isomers of MNI-caged γ-glutamylglycine were based on published protocols for MNI-glycine (Papageorgiou et al., 2011) and for substituted nitroindolines (Papageorgiou and Corrie, 2000). Full experimental details of the synthesis of the two isomers and their structural verification are given in the Supplementary Information (Data sheet 1), together with circular dichroism spectra (Supplementary Figure S1) to confirm the configurations. The photocleavage reaction is outlined in Scheme 1 (below).

## Results

### Time-Course of γ-DGG Concentration Following Photorelease From MNI-Caged γ-DGG

Flashlamp photolysis releases ligand over a large field and it is important for interpretation of results to know the time-course of release and the persistence of released *γ*-DGG in the photolysis region. The release of carboxylate ligands from nitroindoline-caged precursors was shown by nanoseconds laser flash photolysis to have a halftime of 150 ns (Morrison et al., 2002). Thus, on the 0.1 ms timescale relevant for synaptic function, photolysis closely follows the light intensity, the quantity of γ-DGG released and its concentration in the irradiated volume are given by the time integral of the flash. A photodiode was used to monitor light intensity applied by photolysis. The photodiode output (dotted gray traces) and their integral (dashed traces) are shown in Figure 1 for low (300 V, one capacitor, panel **A**) and high energy (three capacitors, panel **B**) flashes. Simultaneous voltage clamp recording in the Purkinje neuron shows transient current (black solid traces) evoked by photolysis; these are discussed below. The γ-DGG concentrations immediately after the flash were calculated from the photolysis efficiency at the flashlamp energy used and the bath concentration of MNI-caged γ-DGG measured by spectrometry at the end of the recording. The photolysis efficiencies were calculated for low or high flash energy from independent experiments in the same microscope as described above in the “Materials and Methods” section and in Supplementary Information.

### Time-Course of Diffusional Loss of Photoreleased γ-DGG From the Irradiated Region

After fast release the concentration of γ-DGG in the region of the Purkinje neuron will decline by diffusional exchange of photoreleased γ-DGG between the irradiated volume, approximately 10 μl, and the bulk bath volume of 1.5 ml. This will determine the concentration of γ-DGG adjacent to synaptic sites with time following photorelease. The highly polar fluorophore HPTS (pyranine) photoreleased in extracellular space has previously been used to measure diffusion within tissues (Xia et al., 1998); it has a diffusion coefficient 330 μm^2^s^−1^, similar to that estimated for synaptic L-glutamate (Nielsen et al., 2004). HPTS was photoreleased from NPE-caged-HPTS releasing fluorophore quickly and uniformly over the field of view (rate of photolysis of 550 s^−1^ at pH 7 following the flash; Jasuja et al., 1999). The fluorescence was monitored after the flash at 5 s intervals with 100 ms camera frames over an aperture placed in a conjugate image plane corresponding to 10 μm (referred to the specimen plane) and corresponding in size to a PC soma. The results are shown for three slices in Figure 1C, giving an average half-time for diffusional loss from the region of the cell of 60 s. In relation to the paired pulse protocol used here the concentration of HPTS over an interval of 50 or 150 ms declined to 90%–95%, and in the 30 s interval between paired stimuli declined to 60%–70% of the concentration released by photolysis associated with the preceding paired pulse stimulation. Rapid mixing of the bath between experimental protocols was by a perfusion tube and remote syringe.

### Transient Inward Current During Photolysis Due to Proton Release

**Figure 2:**
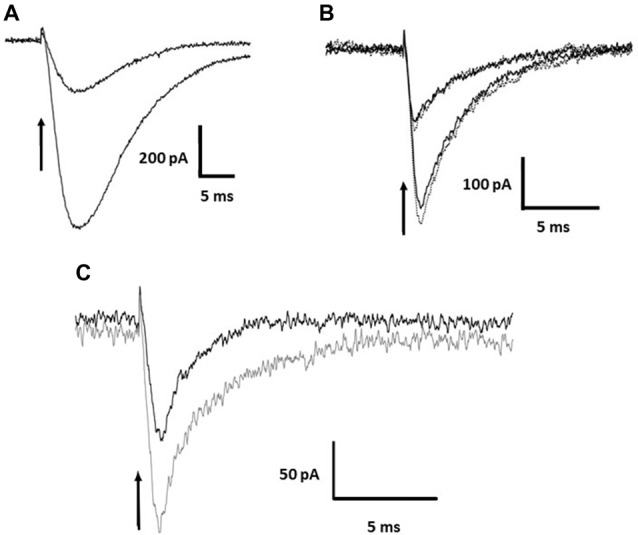
Current evoked by photolysis of MNI-caged *γ*-DGG or MNI-caged *γ*-LGG.** (A)** Currents evoked by photorelease of L-glutamate at 3 μM and 7 μM in a Purkinje cell. **(B)** Currents of similar amplitude evoked by photorelease of 0.5 or 1.0 mM *γ*-DGG, in the absence (solid black traces) or presence (dotted gray traces) of the AMPA/Kainate receptor antagonist NBQX (30 μM). Currents evoked by *γ*-DGG are faster than the glutamate evoked currents and are not affected by the high affinity AMPA receptor competitive antagonist NBQX, indicating that *γ*-DGG is not an agonist of AMPA/Kainate receptors. **(C)** To test for an effect of the protons co-released with *γ*-DGG and *γ*-LGG (see Scheme 1), photorelease of 1.0 mM γ-LGG was compared in saline buffered with 70 mM HEPES (black trace) or 10 mM HEPES (gray trace). High proton buffering reduced the amplitude of the currents. Time of release indicated by the arrow.

The photolysis of MNI-caged γ-DGG shown in Figures 1A,B evokes a transient inward current. Release of 0.5 or 1 mM of either *γ*-DGG (shown in Figures 1A,B, 2B) or *γ*-LGG (not shown) generated transient currents of approximately 0.25 nA and 0.6 nA amplitude that followed closely the light intensity in rise-time, and subsequently declined with a fast milliseconds time-course. As shown in Scheme 1, the photolysis of MNI-caged γ-DGG generates γ-DGG, a proton and a nitrosoindole by-product in stoichiometric proportion. Each of these products could activate the transient inward current observed on photolysis. γ-DGG released at high concentration by fast photolysis might bind and activate AMPA, Kainate or NMDA receptors on a milliseconds timescale, functioning as a weak or partial agonist. By comparison with the *γ*-DGG evoked currents however, photorelease of L-glutamate (3 μM or 7 μM) from MNI-caged L-glutamate to generate similar peak amplitudes evoked currents with much slower onset and decline (Figure 2A), suggesting that activation of AMPA-receptors by *γ*-DGG or *γ*-LGG is not responsible for the fast current. Moreover, photorelease of γ-DGG or γ-LGG at 0.5 mM or 1 mM in presence of NBQX (30 μM, Figure 2B) and D-AP5 (50 μM, data not shown) evoked transient currents with the same amplitudes as control. As a further test for AMPA receptor activation and rapid desensitization, the effect of cyclothiazide (100 μM), a blocker of AMPA receptor desensitization, affected neither amplitude nor time-course of the currents. The data show that γ-DGG is not acting as a weak agonist at AMPA-Kainate receptors nor promoting desensitization, supporting the conclusion from steady-state experiments that it acts as a fast-competitive antagonist (Liu et al., 1999; Wadiche and Jahr, 2001).

A second possibility to account for the transient current may be the activation of a proton-gated channel or proton exchanger by the H^+^ released stoichiometrically with γ-DGG during photolysis, shown in Scheme 1. A first test was to increase the pH buffering from 10 mM HEPES (buffer capacity 5 mM/pH at pH 7.3) to 70 mM, a 7-fold greater equilibrium buffer capacity. The result is illustrated in Figure 2C. The peak amplitude in 70 mM HEPES (black trace) was reduced to 50% of that in 10 mM HEPES (gray trace), and the decay of the current was shortened from 5 ms to 2 ms, indicating a role of protons in the activation of the current. However, the partial inhibition and faster time-course of the currents by high HEPES concentration suggest that the kinetics of buffering by HEPES is too slow to bind protons on the timescale of photorelease.

ASIC channels are known to be present in Purkinje neurons and to respond rapidly to protons (Lingueglia et al., 1997). As a further test of the activation of a proton gated channel, an inhibitor of ASIC channels, IEPA, an analog of amiloride, was tested at the concentration of 30 μM. In two PC the current was inhibited by 30%, however tests of recovery were not possible because the concentration that could be applied was limited by toxicity of the vehicle DMSO. Amiloride itself also produced incomplete inhibition at high concentration, tested at concentrations up to 500 μM. ASIC channels are the most likely candidates for the observed current however known blockers amiloride and derivatives tested here were ineffective and the origin of this conductance will require further pharmacological testing.

Finally, the nitrosoindole by-product generated by the photolysis reaction could in principle be responsible for the transient current. This possibility cannot easily be tested independently since there are no fast-acting reagents available to quench potential oxidative actions. The reducing thiols DTT and glutathione have been shown to react too slowly to be useful in this context (half-time 120 s; Papageorgiou et al., 2008). Evidence from previous experiments however, has suggested that any interference by the nitroso by-products are slow and not on the time-course of the photolysis pulse (Papageorgiou et al., 2011). Thus, it is unlikely that the fast currents are generated by this product of the photolysis reaction.

The evidence suggests that the transient conductance activated upon photolysis of MNI-caged γ-DGG is due to activation of ASIC channels and other conductances, such as electrogenic exchangers, by the protons released stoichiometrically with γ-DGG. Current evoked by proton release during photolysis of MNI-caged ligands has not been demonstrated previously because L-glutamate or other agonists have not been applied with wide-field photolysis at the high concentrations used here for release of γ-DGG.

### Tests for Interference of MNI-Caged *γ*-DGG With Climbing Fiber-Purkinje Cell (CF-PC) Synaptic Transmission

**Figure 3:**
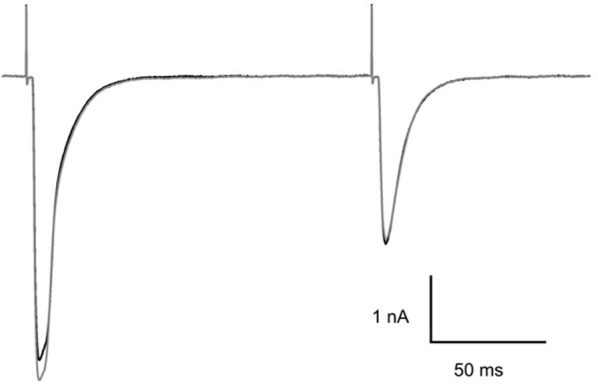
Effect of MNI-caged γ-DGG on CF-PC synaptic transmission. Paired pulse stimulation at 150 ms interval before (black trace) and 5 min after addition of 4.6 mM MNI-caged γ-DGG to the bath (gray trace). Pipette potential −40 mV, 21°C.

Although MNI-caged ligands have been widely used and evaluated since 1999 the concentrations of cage used here are high and were tested for interference with synaptic transmission at CF-PC synapses. The example presented in Figure 3 shows paired CF EPSCs separated by 150 ms before (black trace) and 5 min after addition of MNI-caged γ-DGG to give a concentration of 4.6 mM in the bath (gray trace). The threshold for CF stimulation and the synaptic charge of the first and second EPSC of the pair were compared before and after addition of MNI-caged γ-DGG; data in seven cells with 2.7–5.2 mM caged γ-DGG showed charge in the first EPSC was 0.97 ± 0.023 (SD) of pre-cage control and 0.99 ± 0.018 in the second. No change of stimulation threshold was observed immediately following addition of the cage, although threshold often increased progressively during the experiment.

### Block of Climbing Fiber Transmission at Constant *γ*-DGG Concentration Generated by Photolysis

γ-DGG is a low affinity competitive antagonist that binds to receptors with fast kinetics similar to L-glutamate. Consequently, there is competition between γ-DGG and L-glutamate that reaches equilibrium on the time-scale of the synaptic glutamate concentration transient; this property has been used to estimate changes in the amount of transmitter released from the reduced inhibition by γ-DGG at high glutamate concentration (Liu et al., 1999; Wadiche and Jahr, 2001). The CF-PC synapse shows short-term depression attributed to reduced transmitter release in the second pulse of a pair, resulting in reduced competition and greater fractional block by equal concentrations of γ-DGG in the second compared to the first response (Wadiche and Jahr, 2001). The slow diffusion of γ-DGG from the region of photolysis of MNI-caged-γ-DGG shown above permits estimates of the block at near steady concentration following flash photolysis and may be compared with published steady state data. In experiments to test this, CF were stimulated on a 30 s cycle with pairs of stimuli separated by 40 ms. After acquiring control data over two or more cycles, γ-DGG was released by photolysis coinciding with the first stimulus of a pair. Since γ-DGG diffuses slowly from the region of photolysis the concentration remained high during the second stimulus. The steady state inhibition was calculated by subtraction of the EPSC in γ-DGG from the control followed by normalization to the control EPSC amplitude.

**Figure 4:**
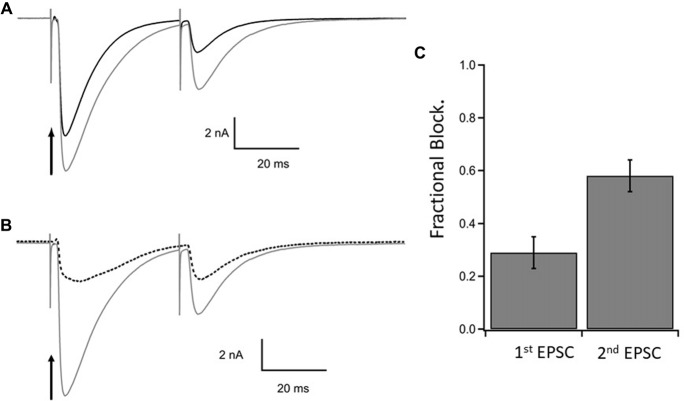
Steady state inhibition of climbing fiber paired pulse EPSCs. **(A)** Control paired EPSCs in gray and test EPSCs in black following photorelease of 1.1 mM γ-DGG at the time of stimulation of the first EPSC. Paired stimulations 40 ms interval. γ-DGG inhibits the second EPSC more than the first. **(B)** The current blocked by γ-DGG (dashed line) was obtained by subtracting test (black trace) from control (gray trace) and is compared to control. **(C)** Fractional block of the peak current of 1st and 2nd EPSC following a flash to release γ-DGG at the first stimulus; data from 10 cells, Mean ± SEM. Range of γ-DGG concentrations released 0.55–1.7 mM. Note that diffusional loss of photoreleased γ-DGG is negligible on this time scale (see Figure 1C). Panels **(B,C)** show that the γ-DGG blocked component is a greater fraction of the control in the second EPSC than the first, indicating a lower competing transmitter concentration in the second EPSC.

The results are illustrated in Figure 4 which shows the EPSCs elicited by a pair of CF stimuli separated by 40 ms in the presence of MNI-caged γ-DGG before (gray trace, control) and after photolysis timed with the first stimulus (black trace). The fraction of current blocked by γ-DGG was obtained by subtracting the current recorded after photolysis from the control, giving the γ-DGG-sensitive current (dotted traces in Figure 4B). Data from 10 cells are summarized in the bar graph Figure 4C. The average fractional block of the first pulse was 0.29 ± 0.06 (SEM) and of the second by 0.60 ± 0.06 (SEM) respectively, measured in Figure 4C at the peak current. The greater block of the second EPSC is consistent with the interpretation that transmitter release and concentration are less in the second pulse than the first during paired pulse depression at CF-PC synapses (Wadiche and Jahr, 2001).

### Timed Inhibition of the CF EPSC by Photoreleased *γ*-DGG: Delayed Release and Sustained Transmitter Presence

**Figure 5:**
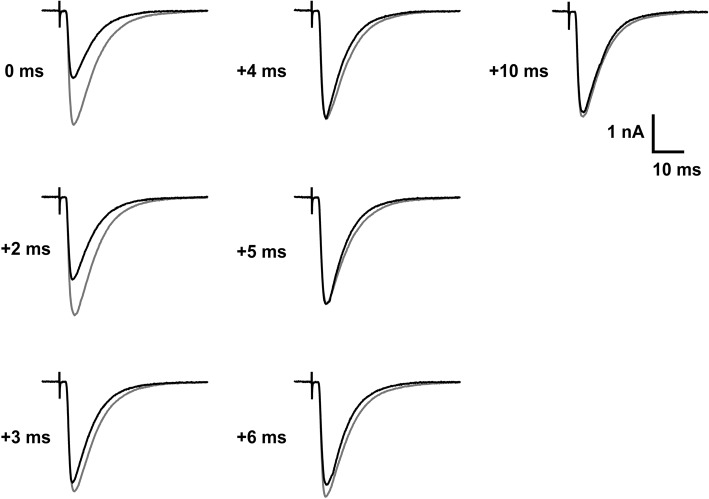
Timed inhibition of CF-PC EPSC by photorelease of γ-DGG. Each panel shows the second EPSCs of a pair separated by 150 ms, the control (gray traces) and 30 s later with photorelease of 0.74 mM γ-DGG (black traces), released in a 1 ms flash from the indicated times relative to the stimulus. The bath contained 5.9 mM MNI-caged γ-DGG. At early times (0 and 2 ms delay from the stimulus) inhibition is comparable to steady-state inhibition. At later times of γ-DGG release (triggered at 3–10 ms) the EPSC approaches the control EPSC peak and only the decay is affected. Age 14 days, −50 mV, 24°C.

**Figure 6:**
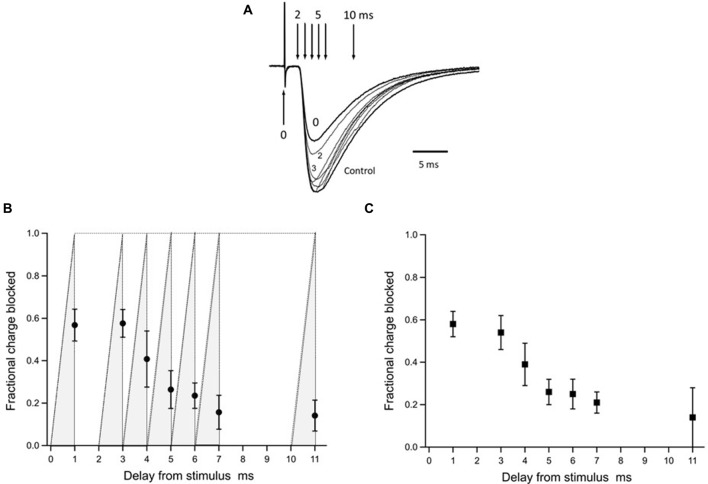
Inhibition by γ-DGG photoreleased during the EPSC: estimating the timing of the synaptic transmitter transient. Panel **(A)** shows the EPSCs presented in Figure 5 superimposed for comparison of the time-course; the record obtained by release of γ-DGG between 0 and 1 ms, coincident with the stimulus, is marked 0; records corresponding to release over 2–3 ms and 3–4 ms post-stimulus are labeled 2 and 3 respectively. Vertical arrows indicate the trigger timing of photolysis pulses releasing γ-DGG as delay times from the stimulus. Panel **(B)** summarizes data from 4 to 8 cells of the fraction of total synaptic charge blocked by γ-DGG. Data are Mean ± SD plotted at the end of the 1 ms photolysis pulse. The integration of the light pulse is indicated by the gray shading for each data point and represents the time-course of γ-DGG concentration increase during the flash, uniform over the microscope field. To obtain the fractional charge blocked by γ-DGG test EPSCs were integrated over 60 ms following the stimulus, subtracted from and normalized to the integral of control EPSCs recorded 30 s earlier. MNI-caged-γ-DGG concentration range 2.7–5.9 mM, γ-DGG released range 0.34–0.74 mM. Panel **(C)** to quantify γ-DGG fractional block late in the EPSC, integrations were made from the time of the flash to 60 ms, subtracted from and normalized to the same period in control. To control for the proton current artifact a record was taken with the flash but without stimulation and subtracted from the test records obtained with stimulation. As a further check the records in **(C)** were integrated from 1 ms after the flash, requiring no subtraction of initial current. Results with or without subtraction were similar. Animal ages 13 days in **(A)**, 13–14 days in **(B,C)**. Temperature 21–24°C.

The fast photorelease of γ-DGG from MNI-caged-γ-DGG was used here to probe transmitter action at postsynaptic receptors during the CF EPSC in order to test the timing and duration of release, and the possible contribution of receptor activation late in the EPSC. The analysis was made on the second of paired EPSCs with 150 ms interval in a 30 s stimulus cycle. The second EPSC was used because the smaller second EPSCs are less affected by poor voltage clamp spatial uniformity. The flashlamp pulse was 1 ms duration (see low intensity flash of Figure 1A) and delayed by 0–10 ms with respect to the CF stimulation. The protocol and results are illustrated by the records shown in Figure 5, traces in gray are controls and in black after photorelease of 0.74 mM γ-DGG at the times indicated. Timing was set with respect to the stimulus, the γ-DGG concentration rising with the integral of light intensity over 1 ms from the time indicated, as shown in Figure 1A and Figure 6B. Release prior to transmitter binding to postsynaptic receptors is predicted to produce block similar to the steady state block shown for the second EPSC in Figure 4. Release of γ-DGG after transmitter release will produce inhibition if transmitter is present and postsynaptic receptors are available.

Each panel of Figure 5 shows the EPSC recorded with photolysis triggered at the time indicated following CF stimulation (black traces), superimposed with the control EPSC recorded 30 s earlier (gray traces). The results show that γ-DGG release with 0–1 ms or 2–3 ms delay from the stimulus resulted in a reduced peak amplitude, similar to the steady state inhibition of 60% described in Figure 4C. At longer delays γ-DGG released over 4–5 ms, 5–6 ms, 6–7 ms from the stimulus showed the same peak EPSC as control but a faster decay. γ-DGG released in the interval 3–4 ms reduced the peak amplitude of the EPSC much less than at earlier times and speeds the decline, indicating competition with transmitter during this interval. The records are superimposed in Figure 6A to permit comparison of changes in the EPSC waveform.

The block by γ-DGG was quantified by integrating the EPSC to obtain the total synaptic charge in the absence and presence of γ-DGG release. The charge blocked by γ-DGG was obtained by subtracting the integrals and then normalizing to control so as to permit comparisons among experiments. The data were integrated over the whole EPSC (from the CF stimulus for 60 ms; data in Figure 6B) or from the time of the flash (to 60 ms post-stimulus; Figure 6C).

The data from eight cells are summarized in Figure 6B as the fraction of synaptic charge blocked by γ-DGG photorelease at different times delayed with respect to the stimulus. The fraction of EPSC charge blocked by γ-DGG is given for each delay as Mean ± SD of 4–8 determinations plotted at the end of the flash. The γ-DGG concentration range was 0.34–0.74 mM. The results show that release of γ-DGG in the intervals 0–1 ms and 2–3 ms after the stimulus produced block at similar levels, both with mean 60%, and similar to the steady-state block of the second EPSC shown in Figure 4C. This indicates that receptor activation by transmitter release had not started after 3 ms from the stimulus. γ-DGG released over the interval 3–4 ms after the stimulus produced a reduced mean block of 40%, indicating competition with transmitter for a shorter time in this interval. Also, the SD is larger, approximately double that seen at other time points, indicating jitter in the timing of transmitter release between measurements. Later, with γ-DGG release at 4–5 ms and 5–6 ms, the block is smaller and less variable. The data therefore suggest that glutamate binding of postsynaptic receptors in competition with photoreleased γ-DGG occurs mainly at 3–4 ms following CF stimulation, a time corresponding to the initial rise of the EPSC. A second observation from Figure 6B (seen also in Figures 5, 6A) is the continued inhibition by γ-DGG when released at 5–6 ms, close to the peak EPSC, or at longer delays. This suggests that transmitter is available to bind free receptors late in the EPSC. As a better measure of γ-DGG block at late times the data were expressed as the fraction of EPSC charge blocked relative to control by integrating and comparing the EPSC charge only over the period following the flash. The results plotted in Figure 6C show a substantial fraction, average 23%, of the EPSC charge is blocked by γ-DGG released at 6 ms and later times post-stimulus. Although the change in EPSC is small at 5–6 ms and later, the synaptic charge is significant and the observations nonetheless indicate the presence of transmitter at the later time points. The results imply receptor availability and reactivation by transmitter, due either to delayed release or persistence in the synapse. It may be noted that late in the EPSC the fractional block at low transmitter concentration is expected to be constant in absence of competition, simply related to the concentration of γ-DGG and its equilibrium constant for receptor binding (Liu et al., 1999).

## Discussion

MNI-caged-γ-DGG was developed as a tool to probe the activation of postsynaptic receptors during transmission at glutamatergic synapses, to investigate the roles of spillover or volume transmission and the functional independence of synaptic sites. A main purpose of the experiments reported was the verification of MNI-caged-γ-DGG as a probe suitable for testing receptor activation in synaptic and parasynaptic transmission. The CF-PC synapse was studied as a single connection permitting an assessment of the new cage. No detrimental effects of the cage itself were seen on CF-PC transmission, as judged by threshold for stimulation, synaptic properties or short-term plasticity tested by paired pulse depression, at concentrations up to 5 mM of MNI-caged γ-DGG or MNI-caged γ-LGG. The cages synthesized and purified with the methods described above and in Supplementary Information were soluble in physiological saline at 20 mM concentration, enabling use from stock solutions. It is also important that there was no loss of chiral specificity in the synthesis, as shown by the results of CD spectrometry (see SI, Supplementary Figure S1). The MNI cages have well tested properties of hydrolytic stability (Papageorgiou et al., 1999), sub-microsecond release (Morrison et al., 2002) and good pharmacological properties at glutamate synapses (Canepari et al., 2001; Trigo et al., 2009; Palma-Cerda et al., 2012). The ability to use high concentrations without detrimental effects on excitability or transmitter release, the known efficiency with 405 nm laser photolysis of the MNI-cages (Trigo et al., 2009), and the time-resolution of photolysis suggest MNI-caged- γ-DGG is a useful synaptic probe.

To our knowledge, this is the first study that tests the fast release of a fast equilibrating antagonist at AMPA-Kainate and NMDA receptors and we were interested to know whether transient activation of receptors might be apparent under these release conditions. There was no partial agonist activity of γ-DGG seen when applied at mM concentration by photorelease on a sub-millisecond timescale. This indicates that the receptor binding of γ-DGG does not induce conformation changes that result in transient channel gating and desensitization. It is consistent with the equilibrium data of Liu et al. (1999) that γ-DGG has properties of a competitive receptor antagonist, apparently showing no efficacy for channel gating. We also tested the ability of MNI-caged-LGG to block CF-PC synapses upon photolysis, the results confirming that γ-LGG is much less potent than γ-DGG at similar high concentrations.

The immediate physiological results presented here, an analysis of the EPSC at the CF-PC synapse, show two interesting aspects. The first is a long delay of 3–4 ms from the stimulus to the first evidence of receptor activation. Since the stimulus site is close, within 50 μm of the Purkinje soma, the conduction delay is predicted to be short, less than 100 μs. At synapses where the delay from presynaptic spike to postsynaptic activation has been measured it is 0.5–1 ms at room temperature (Katz and Miledi, 1965; Forsythe, 1994). The delay from stimulus to release here is therefore approximately 1 ms longer than can be accounted for by synaptic delay and conduction velocity in small myelinated fibers. It may point to other processes such as additional conduction delays in the CF terminals that may delay release.

The second finding is a late component of receptor activation occurring after the major period of activation at 3–4 ms post-stimulus. Vesicular release is known to continue at a reduced rate for several milliseconds after the peak release at about 1 ms (Katz and Miledi, 1965; Trigo et al., 2012) and may contribute a late component. An additional factor contributing to the activation of postsynaptic current late in the EPSC may be persistence of glutamate in the synapse. The CF terminals are protected by Bergmann glial processes and glutamate transporters at high density on both the PC and the glial membranes which will quickly buffer and take up the transmitter. The experiments here were done at 21–24°C and both release and uptake will be slower compared with physiological temperature (Auger and Attwell, 2000). The relative contributions of the two mechanisms are not addressed here and will be better examined at physiological temperature.

The possibility that γ-DGG acts at presynaptic glutamate receptors to modify transmitter release as well as postsynaptically has not been excluded. Presynaptic effects were tested by Wadiche and Jahr (2001) by monitoring transporter currents, with the finding that these were unaffected by addition of 2 mM γ-DGG. However, the transporter currents in their study were isolated by a high concentration of NBQX which would mask an effect of γ-DGG on presynaptic AMPA-K receptors. While ruling out direct effects of γ-DGG on release, the possibility of block by γ-DGG of receptor mediated modulation of transmission by presynaptic AMPA-K or NMDAR is difficult to test. There are, however, no reports of presynaptic AMPAR or NMDAR at climbing fiber terminals.

We have noted previously a selectivity of γ-DGG for Bergmann glial AMPAR with about 10-fold higher affinity than Purkinje AMPAR (Bellamy and Ogden, 2005). This may relate to the different receptor composition, GluA1 and GluA4 in Bergmann glia and GluA1, GluA2 and GluA3 in Purkinje neurons, and the presence of auxiliary proteins, particularly TARP γ2. Thus, there is the additional possibility of time resolved experiments with pharmacological specificity for AMPA receptors of different subunit and auxiliary protein composition by the photorelease of γ-DGG at low or high concentrations to distinguish receptor types pharmacologically. Further, evidence has been presented that kinetic parameters and functional affinity of AMPAR for transmitter L-glutamate differs among TARP vs. non-TARP associated receptors (Zhang et al., 2014; Carbone and Plested, 2016; Devi et al., 2016). If the affinity for L-glutamate differs among AMPA-R of different compositions and gating mode, the estimates of transmitter concentration with steady-state application of γ-DGG will be modified due to competition and to differences in affinity.

An important result obtained here in the context of uncaging methodology was the activation in Purkinje cells of a small fast inward current gated by mM release of protons. Protons are released during nitrobenzyl and nitroindoline photolysis stoichiometrically with the ligand, however usually the concentrations are low and, in the case of local laser photolysis, rapidly dissipated by diffusion. The high concentrations released over a large volume here are unusual. The data shown in Figure 2 indicate that even raising the buffer capacity from 10 mM to 70 mM HEPES was not effective in completely suppressing the current; even if the protonation rate is high in aqueous solution, estimated as 10^10^ L.mole^−1^.s^−1^, the pK of 7.3 indicates a deprotonation rate in the milliseconds range for HEPES. Further, the normal bicarbonate-CO_2_ buffer is known to have kinetics on a second timescale and will be ineffective on a millisecond timescale. These considerations apply also to the strong alkalinization produced by photolysis of DMnitophen and NPE-EGTA to release Ca intracellularly (Trigo et al., 2012). The origin of the inward current activated by protons here is not clear. ASIC channels are known to be present in Purkinje neurons and to respond rapidly to protons (Lingueglia et al., 1997), however known blockers amiloride and derivatives tested here were ineffective. Other possibilities are electrogenic transporters with coupled proton flux or proton permeabilities which may be tested pharmacologically.

The time resolution in the experiments reported here is determined by the 1 ms duration of the flashlamp discharge, measured for each record to determine the precise timing of γ-DGG release but longer than the 0.1 ms limit imposed by equilibration in receptor binding by γ-DGG (Liu et al., 1999; Wadiche and Jahr, 2001). The flashlamp provides widefield photolysis and was necessary because of the large field of CF dendritic innervation. At small synaptic contacts laser photolysis over a smaller area will give time resolution of 0.1 ms with MNI-caged γ-DGG at individual synapses with the methods described by Trigo et al. (2009). As well as improved time resolution, the extent of photolysis is greater with laser activation, permitting greater economy with the cage and better control of the concentration released.

Recently the time resolution of tethered genetically encoded fluorescent glutamate reporters has been improved to the milliseconds level (Helassa et al., 2018) providing another way to investigate transmitter concentration profiles at single synapses. The two approaches are complementary, the present method more readily applicable because there is no requirement for transgene or viral labeling. Combining the two approaches is unlikely because of the strong bleaching action of near-UV on the GFP reporter fluorescence.

Although MNI cages have properties of speed, stability and pharmacological inertness it is generally known that they are very inefficient in two-photon photolysis, requiring high concentrations and high intensities, releasing a few percent of the cage concentration at non-toxic intensities. The two-photon cross-section measured by us and in other studies is 0.05 GM, whereas the cross-section required for 50% photolysis in the laser spot is two orders larger at the threshold for non-toxic exposure, an average power of 5 mW. Phototoxicity of ultra-short pulsed irradiation is seen presynaptically, characterized by aberrant release at average powers greater than 5 mW (Hopt and Neher, 2001; Kiskin et al., 2002). Two-photon excitation of MNI-caged-γ-DGG to release useful concentrations of γ-DGG would be phototoxic and too localized to be useful in testing distributed transmitter release.

In conclusion, we present the synthesis and evaluation of a fast-caged antagonist MNI-caged-γ-DGG and the methods to apply it in the analysis of synaptic transmission in this case with wide-field photolysis at the CF-PC synapse. The results define the time-window of receptor activation by transmitter, present evidence for a delay in transmission or release, and persistence of receptor activation by transmitter late in the EPSC.

## Author Contributions

FP-C, GP, BB, CA and DO contributed to the conception and design of the study. GP synthesized and characterized reagents. FP-C and DO performed experiments and analyzed data. DO wrote the first draft of the manuscript. All authors contributed to manuscript revision, read and approved the submitted version.

## Conflict of Interest Statement

The authors declare that the research was conducted in the absence of any commercial or financial relationships that could be construed as a potential conflict of interest.

## References

[B1] Auger C. Attwell D. (2000). Fast removal of synaptic glutamate by postsynaptic transporters. Neuron 28, 547–558. 10.1016/s0896-6273(00)00132-x 11144363

[B2] Beato M. (2008). The time course of transmitter at glycinergic synapses onto motoneurons. J. Neurosci. 28, 7412–7425. 10.1523/JNEUROSCI.0581-08.2008 18632945 PMC2615222

[B3] Bellamy T. C. Ogden D. (2005). Short-term plasticity of Bergmann glial cell extrasynaptic currents during parallel fiber stimulation in rat cerebellum. Glia 52, 325–335. 10.1002/glia.20248 16078233

[B4] Canepari M. Nelson L. Papageorgiou G. Corrie J. E. T. Ogden D. (2001). Photochemical and pharmacological evaluation of 7-nitroindolinyl-and 4-methoxy-7-nitroindolinyl-amino acids as novel, fast caged neurotransmitters. J. Neurosci. Methods 112, 29–42. 10.1016/s0165-0270(01)00451-4 11640955

[B5] Carbone A. L. Plested A. J. (2016). Superactivation of AMPA receptors by auxiliary proteins. Nat. Commun. 8:10178. 10.1038/ncomms10178 26744192 PMC4729862

[B6] Clements J. D. Lester R. A. Tong G. Jahr C. E. Westbrook G. L. (1992). The time course of glutamate in the synaptic cleft. Science 258, 1498–1501. 10.1126/science.1359647 1359647

[B7] Coddington L. T. Nietz L. K. Wadiche J. I. (2014). The contribution of extrasynaptic signalling to cerebellar information processing. Cerebellum 13, 513–520. 10.1007/s12311-014-0554-7 24590660 PMC4077919

[B8] Davies J. Watkins J. C. (1981). Differentiation of kainate and quisqualate receptors in the cat spinal cord by selective antagonism with γ-D (and L)-glutamylglycine. Brain Res. 206, 172–177. 10.1016/0006-8993(81)90111-6 6258721

[B9] Devi S. P. Howe J. R. Auger C. (2016). Train stimulation of parallel fiber to Purkinje cell inputs reveals two populations of synaptic responses with different receptor signatures. J. Physiol. 594, 3705–3727. 10.1113/jp272415 27094216 PMC4929331

[B10] Forsythe I. D. (1994). Direct patch recording from identified presynaptic terminals mediating glutamatergic EPSCs in the rat CNS, *in vitro*. J. Physiol. 479, 381–387. 10.1113/jphysiol.1994.sp020303 7837096 PMC1155757

[B11] Francis A. A. Jones A. W. Watkins J. C. (1980). Dipeptide antagonists of amino acid-induced and synaptic excitation in the frog spinal cord. J. Neurochem. 35, 1458–1460. 10.1111/j.1471-4159.1980.tb09025.x 6969293

[B12] Helassa N. Dürst C.-D. Coates C. Kerruth S. Arif U. Schulze C.. (2018). Ultrafast glutamate sensors resolve high-frequency release at Schaffer collateral synapses. Proc. Natl. Acad. Sci. U S A 115, 5594–5599. 10.1073/pnas.1720648115 29735711 PMC6003469

[B13] Hopt A. Neher E. (2001). Highly nonlinear photodamage in two-photon fluorescence microscopy. Biophys. J. 80, 2029–2036. 10.1016/s0006-3495(01)76173-5 11259316 PMC1301392

[B14] Jasuja R. Keyoung J. Reid G. P. Trentham D. R. Khan S. (1999). Chemotactic responses of *Escherichia coli* to small jumps of photoreleased L-aspartate. Biophys. J. 76, 1706–1719. 10.1016/s0006-3495(99)77329-7 10049350 PMC1300146

[B15] Kanazawa A. Kakimoto Y. Nakajima T. Shimizu H. Takesada M. Sano I. (1965). Isolation and identification of γ-L-glutamylglycine from bovine brain. Biochim. Biophys. Acta 97, 460–464. 10.1016/0304-4165(65)90157-1 14323591

[B16] Katz B. Miledi R. (1965). The effect of temperature on the synaptic delay at the neuromuscular junction. J. Physiol. 181, 656–670. 10.1113/jphysiol.1965.sp007790 5880384 PMC1357674

[B17] Kiskin N. I. Chillingworth R. McCray J. A. Piston D. Ogden D. (2002). The efficiency of two-photon photolysis of a “caged” fluorophore, o-1–(2-nitrophenyl)ethylpyranine, in relation to photodamage of synaptic terminals. Eur. Biophys. J. 30, 588–604. 10.1007/s00249-001-0187-x 11908850

[B18] Lingueglia E. de Weille J. R. Bassilana F. Heurteaux C. Sakai H. Waldmann R.. (1997). A modulatory subunit of acid sensing ion channels in brain and dorsal root ganglion cells. J. Biol. Chem. 272, 29778–29783. 10.1074/jbc.272.47.29778 9368048

[B19] Liu G. Choi S. Tsien R. W. (1999). Variability of neurotransmitter concentration and nonsaturation of postsynaptic AMPA receptors at synapses in hippocampal cultures and slices. Neuron 22, 395–409. 10.1016/s0896-6273(00)81099-5 10069344

[B20] Morrison J. Wan P. Corrie J. E. T. Papageorgiou G. (2002). Mechanisms of photorelease of carboxylic acids from 1-acyl-7-nitroindolines in solutions of varying water content. Photochem. Photobiol. Sci. 1, 960–969. 10.1039/b206155d 12661593

[B21] Nielsen T. A. DiGregorio D. A. Silver R. A. (2004). Modulation of glutamate mobility reveals the mechanism underlying slow-rising AMPAR EPSCs and the diffusion coefficient in the synaptic cleft. Neuron 42, 757–771. 10.1016/j.neuron.2004.04.003 15182716

[B22] Palma-Cerda F. Auger C. Crawford D. J. Hodgson A. C. C. Reynolds S. J. Cowell J. K.. (2012). New caged neurotransmitter analogs selective for glutamate receptor sub-types based on methoxynitroindoline and nitrophenylethoxycarbonyl caging groups. Neuropharmacology 63, 624–634. 10.1016/j.neuropharm.2012.05.010 22609535

[B23] Papageorgiou G. Beato M. Ogden D. (2011). Synthesis and photolytic evaluation of a nitroindoline-caged glycine with a side chain of high negative charge for use in neuroscience. Tetrahedron 67, 5228–5234. 10.1016/j.tet.2011.05.045

[B24] Papageorgiou G. Corrie J. E. T. (2000). Effects of aromatic substituents on the photocleavage of 1-acyl-7-nitroindolines. Tetrahedron 56, 8197–8205. 10.1016/s0040-4020(00)00745-6

[B25] Papageorgiou G. Ogden D. Barth A. Corrie J. E. T. (1999). Photorelease of carboxylic acids from 1-acyl-7-nitroindolines in aqueous solution: rapid and efficient photorelease of L-glutamate. J. Am. Chem. Soc. 121, 6503–6504. 10.1021/ja990931e

[B26] Papageorgiou G. Ogden D. Corrie J. E. T. (2008). An antenna-sensitised 1-acyl-7-nitroindoline that has good solubility properties in the presence of calcium ions and is suitable for use as a caged L-glutamate in neuroscience. Photochem. Photobiol. Sci. 7, 423–432. 10.1039/b800683k 18385884

[B27] Scimemi A. Beato M. (2009). Determining the neurotransmitter concentration profile at active synapses. Mol. Neurobiol. 40, 289–306. 10.1007/s12035-009-8087-7 19844813 PMC2777263

[B28] Szapiro G. Barbour B. (2007). Multiple climbing fibers signal to molecular layer interneurons exclusively via glutamate spillover. Nat. Neurosci. 10, 735–742. 10.1038/nn1907 17515900

[B29] Szapiro G. Barbour B. (2009). Parasynaptic signalling by fast neurotransmitters: the cerebellar cortex. Neuroscience 162, 644–655. 10.1016/j.neuroscience.2009.03.077 19358875

[B30] Trigo F. F. Corrie J. E. T. Ogden D. (2009). Laser photolysis of caged compounds at 405 nm: photochemical advantages, localisation, phototoxicity and methods for calibration. J. Neurosci. Methods 180, 9–21. 10.1016/j.jneumeth.2009.01.032 19427524

[B31] Trigo F. F. Sakaba T. Ogden D. Marty A. (2012). Readily releasable pool of synaptic vesicles measured at single synaptic contacts. Proc. Natl. Acad. Sci. U S A 109, 18138–18143. 10.1073/pnas.1209798109 23074252 PMC3497746

[B32] Wadiche J. I. Jahr C. E. (2001). Multivesicular release at climbing fiber-Purkinje cell synapses. Neuron 32, 301–313. 10.1016/s0896-6273(01)00488-3 11683999

[B33] Xia P. Bungay P. M. Gibson C. C. Kovbasnjuk O. N. Spring K. R. (1998). Diffusion coefficients in the lateral intercellular spaces of Madin-Darby canine kidney cell epithelium determined with caged compounds. Biophys. J. 74, 3302–3312. 10.1016/s0006-3495(98)78037-3 9635784 PMC1299671

[B34] Zhang W. Devi S. P. Tomita S. Howe J. R. (2014). Auxiliary proteins promote modal gating of AMPA- and kainate-type glutamate receptors. Eur. J. Neurosci. 39, 1138–1147. 10.1111/ejn.12519 24712993 PMC4311398

