# Supplementary material for: Photolysis of a Caged, Fast-Equilibrating Glutamate Receptor Antagonist, MNI-Caged γ-D-Glutamyl-Glycine, to Investigate Transmitter Dynamics and Receptor Properties at Glutamatergic Synapses
Source: Front Cell Neurosci. 2018 Dec 5;12:465. doi: 10.3389/fncel.2018.00465 (PMC6300705; doi:10.3389/fncel.2018.00465)
Supplement: Supplementary file 1 [file Data_Sheet_1.doc]

**Supplementary Information.**

**Photolysis of a caged, fast-equilibrating glutamate receptor antagonist, MNI- *γ-*D-glutamyl-glycine, to investigate transmitter dynamics and receptor properties at glutamatergic synapses.**

Francisco Palma-Cerda^1^, George Papageorgiou^2^, Boris Barbour^3^, Celine Auger^1^ and David Ogden^1^

1 Brain Research Laboratory, CNRS UMR8118 Université Paris Descartes, Paris.

2 Francis Crick Institute, London.

3 Institute for Biology IBENS, Ecole Normale Supérieure, Paris.

**General Details**. Organic solvents were dried over anhydrous Na_2_SO_4_ and evaporated under reduced pressure. ^1^H and ^13^C NMR spectra were determined on Bruker Avance III HD Nanobay 400 MHz spectrometer in CDCl_3_ solution with TMS as internal reference, unless otherwise specified. Electrospray high resolution mass spectra were carried out by MEDAC Ltd., Surrey, UK. Merck 9385 silica gel was used for flash chromatography. Analytical HPLC was performed on a Merck Lichrospher RP8 column (250 × 4 mm) column with a mobile phase of 25 mM Na phosphate pH 6 - acetonitrile (10:3 v/v) at a flow rate of 1.5 mL min^-1^. Preparative HPLC was performed on a 2 × 30 cm with Waters C_18_ packing material, and a flow rate of 2.5 mL min^-1^. Detection for both analytical and preparative HPLC was at 254 nm. Photolysis experiments were performed in a Rayonet RPR-100 photochemical reactor fitted with 16 × 350 nm lamps. Note that the *R* and *S* descriptors of stereochemistry correspond to the D and L isomers respectively.

**(*R*)-*tert*-Butyl 2-[(*tert*-butoxycarbonyl)amino]-5-{[2-(4-methoxy-7-nitroindolin-1-yl)-2-oxoethyl]amino}-5-oxopentanoate 1**

To a solution of 1-(2-aminoacetyl)-4-methoxy-7-nitroindoline (469 mg, 1.72 mmol), prepared as previously described, (Papageorgiou, Beato and Ogden, 2011) in dry MeCN (40 mL) was added DMAP (0.64 g, 5.2 mmol), *N-tert*-Boc-D-glutamic acid α-*tert*-butyl ester (0.64 g, 2.1 mmol) and 1-(3-dimethylaminopropyl)-3-ethylcarbodiimide hydrochloride (0.46 g, 2.4 mmol). The mixture was stirred under nitrogen at room temperature for 18 h, then evaporated. The residue was taken up in EtOAc (100 mL) and washed successively with 0.5 M aq. HCl, saturated aq. NaHCO_3_ and brine, dried and evaporated to a viscous oil. Flash chromatography [EtOAc-hexanes (4:1)], followed by trituration with ether, gave the *title compound* (0.85 g, 92%), as pale solid, mp 88-90 ˚C; ^1^H NMR (400 MHz, CDCl_3_): *δ* 7.74 (1H, d, *J* = 9.2 Hz, 6-H), 6.66 (1H, d, *J* = 9.2 Hz, 5-H), 6.63 (1H, br s, CH_2_N*H*), 5.19 (1H, d, *J* = 8.2 Hz, N*H*Boc), 4.23 (2H, t, *J* = 8.2 Hz, 2-H), 4.20 (2H, d, *J* = 4.6 Hz, C*H_2_*NH), 4.10-4.20 (1H, m, CH), 3.90 (3H, s, OCH_3_), 3.10 (2H, t, *J* = 8.0 Hz, 3-H), 2.22-2.38 (2H, m, COC*H*_2_), 2.07-2.19 and 1.80-1.94 (2H, 2 × m, CH_2_), 1.43 [9H, s, (CH_3_)_3_], 1.41 [9H, s, (CH_3_)_3_]. HRMS (ESI): Calcd for (C_25_H_36_N_4_O_9_ + Na)^+^: 559.2380. Found: 559.2381.

**(*S*)-*tert*-Butyl 2-[(*tert*-butoxycarbonyl)amino]-5-{[2-(4-methoxy-7-nitroindolin-1-yl)-2-oxoethyl]amino}-5-oxopentanoate 2**

This material was prepared as above, but substituting *N*-*tert*-Boc-L-glutamic acid α-tert-butyl ester for the L-isomer. The NMR spectrum was identical to that reported above and mass spectrometry as above gave m/z 559.2390.

**(*R*)-2-Amino-5-{[2-(4-methoxy-7-nitroindolin-1-yl)-2-oxoethyl]amino}-5-oxopentanoic acid (MNI-caged γ-D-glu-gly) 3.**

Compound **1** (335 mg, 0.6 mmol) was dissolved in anhydrous trifluoroacetic acid (10 mL) and kept for 1 h at room temperature in the dark. The TFA was evaporated and the remaining gum was dissolved in ice-cold water (20 mL) and quickly adjusted to pH 7.0 with 1 M aq. NaOH. The mixture, which contained some gummy material, was extracted with ether (3 × 30 mL) and the aqueous solution was lyophilised and redissolved in 25 mM Na phosphate, pH 6.0 (40 mL). Analysis by HPLC showed a major peak, *t*_R_ 3.5 min. The solution was pumped onto the preparative reverse-phase HPLC column that had been equilibrated in 25 mM Na phosphate, pH 6 and the column was eluted with the same solvent for 45 min, then with water for 1 h and finally with water-acetonitrile (5:1 v/v). Fractions containing the product **3** were analysed by HPLC as described above, and relevant fractions were combined and lyophilised. The dry powder was redissolved in water, passed through a 0.2 μm filter and the concentration (19.6 mM) was determined by UV-vis spectroscopy, based on ε_322_ 4800 M^-1^cm^-1^ as previously described Papageorgiou and Corrie, 2000). Aliquots were stored at -20 ˚C. ^1^H NMR (400 MHz, D_2_O, acetone ref.: *δ* 7.81 (1H, d, *J* = 9.1 Hz, 6-H), 6.93 (1H, d, *J* = 9.1 Hz, 5-H), 4.31 (2H, t, *J* = 7.9 Hz, 2-H), 4.24 (1H, s, COC*H*_2_NH), 3.96 (3H, s, OCH_3_), 3.71 (1H, t, *J* = 6.0 Hz, C*H*CO_2_(NH_3_), 3.13 (2H, t, *J*= 7.9 Hz, 3-H), 2.50 (2H, t, *J* = 7.6 Hz, COC*H*_2_CH_2_), 2.13 (2H, dt, *J* = 7.6 Hz, (COCH_2_C*H*_2_). ^13^C NMR (100 MHz, D_2_O, acetone ref.): *δ* 175.3 (C_q_, CO_2_^-^), 169.5 (C_q_, C=O), 159.5 (C_q_, Ar), 135.3 (C_q_, Ar), 134.4 (C_q_, Ar), 125.5 (CH, Ar), 123.8 (C_q_, Ar), 108.2 (CH, Ar), 56.1 (OCH_3_), 54.3 (CH), 49.6 (CH_2_), 42.6 (CH_2_), 31.2 (CH_2_), 30.2 (CH_2_), 26.0 (CH_2_). HRMS (ESI): Calcd for (C_16_H_20_N_4_O_7_ + H)^+^ 381.1410. Found: 381.1423.

**(S)-2-Amino-5-{[2-(4-methoxy-7-nitroindolin-1-yl)-2-oxoethyl]amino}-5-oxopentanoic acid (MNI-caged γ-L-glu-gly) 4**

This material was prepared identically to the (*R*)**-**isomer and stored at 25 mM concentration. The NMR spectra were identical to that reported above, and HRMS (ESI) gave a molecular ion at 381.1415.


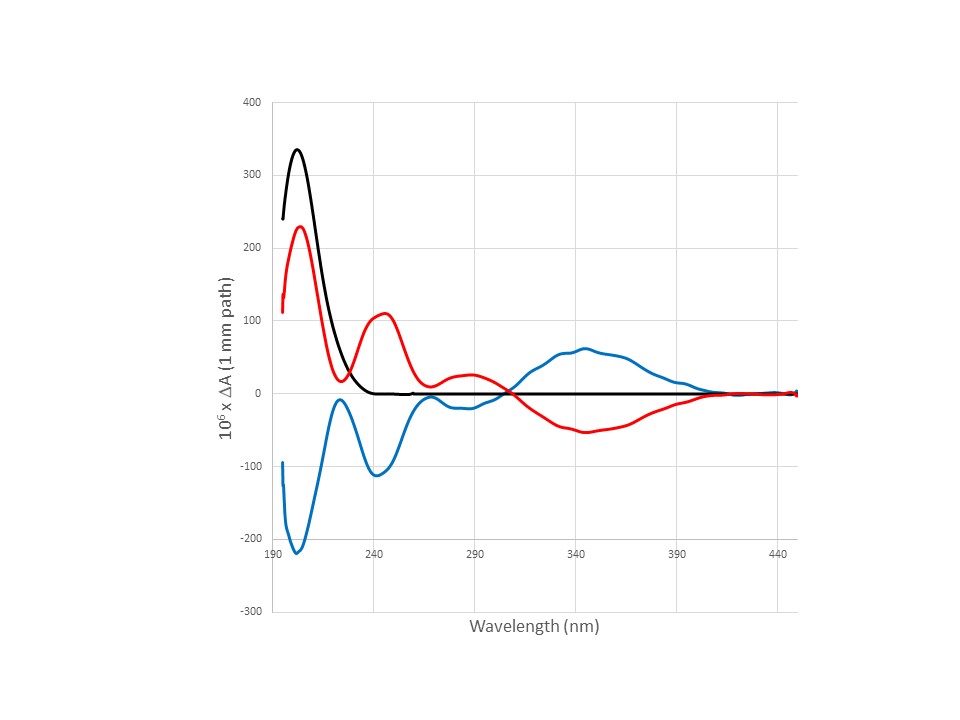


Fig S1. CD spectrum of MNI-caged γ-DGG **3** (Blue), MNI-caged γ-LGG **4** (Red) and L-glutamate (Black). Cage concentrations at approx. 2.5 mM (determined by UV-vis molar absorption coeff 4800 M^-1^cm-^1^ at 322 nm) scaled to L-glutamate 3.5 mM. 10 mM Tris buffer pH 7.0

**CD spectra** were recorded on a Jasco J-815 spectropolarimeter fitted with a cell holder thermostatted at 20 ˚C by a CDF-426S Peltier unit. All CD measurements were made in fused silica cuvettes with 1-mm path length (Hellma, Jena, Germany). The spectra were typically recorded with 0.1-nm resolution and baseline corrected by subtraction of the appropriate buffer spectrum.

**Widefield flashlamp photolysis.**


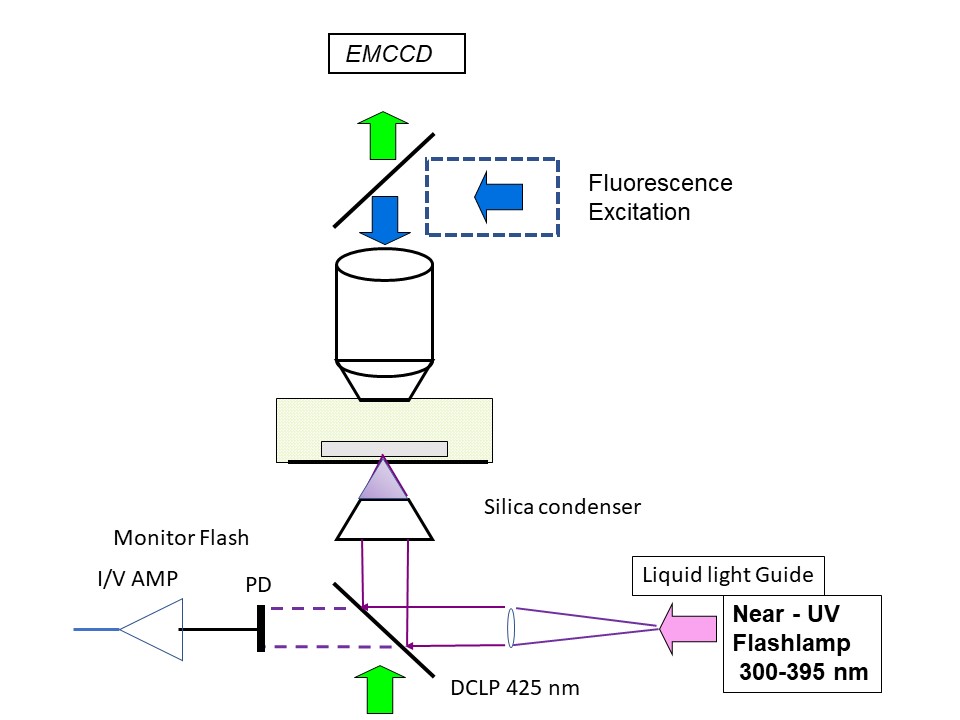


Fig S2.

Diagram showing the optical arrangement for widefield flashlamp photolysis. Flash light from a xenon arc flashlamp (Rapp Optoelectronic, Hamburg) was applied through the condenser to avoid the strong absorption by cage in the bath solution with epi-illumination (OD approx 2 at mM MNI cage concentrations). A silica condenser was used. The flash was SP filtered at 395 nm, coupled through a 3 mm liquid light guide, and after collimation and reflection from a 425 nm DCLP dichroic was focused at the top of the slice. The flash profile was monitored with a silicon photodiode and I/V amplifier from leakage through the dichroic. Transmission through the slice was measured at 360/10 nm as 44% at 12 DAB, 40% at 15 DAB, 32% at 20 DAB. Light output was monitored before the condenser with a photodiode and I/V amplifier to give flash intensity during experiments. The lamp was used at full output (3 capacitors, 300 V 72 mJ/flash at the LLG exit, 1.5 ms duration) or at 50% output (1 capacitor 300 V, 1 ms flash) producing a shorter pulse (see main text Fig.1) attenuated with ND filters as required. Photolysis was calibrated in the microscope with the caged fluorophore NPE-HPTS (50 – 100 μM; Tocris, UK) prepared as small aqueous vesicles (5-20 micron diam, in 10 mM Na Borate pH 9) suspended in Sylgard 184 (Dow Corning). The fraction of cage converted per flash was calculated from the fraction of fluorescence released with each pulse as described by Canepari et al., (2001) and by Trigo et al. (2009). Photolysis conversion/flash measured simultaneously in several vesicles was uniform over the field of view of 200 microns**.**

**SI References**:

Canepari M, Nelson L, Papageorgiou G, Corrie JET, Ogden D. (2001) Photochemical and pharmacological evaluation of 7-nitroindolinyl-and 4-methoxy-7-nitroindolinyl-amino acids as novel, fast caged neurotransmitters. J Neurosci Methods. 112(1):29-42.

Papageorgiou G, Beato M, Ogden D. (2011) Synthesis and photolytic evaluation of a nitroindoline-caged glycine with a side chain of high negative charge for use in neuroscience. Tetrahedron. 67(29):5228-5234.

Papageorgiou G, Corrie JET. (2000) Effects of aromatic substituents on the photocleavage of 1-acyl-7-nitroindolines. Tetrahedron. 56(41):8197-8205.

Trigo FF, Corrie JET, Ogden D. (2009) Laser photolysis of caged compounds at 405 nm: photochemical advantages, localisation, phototoxicity and methods for calibration. J Neurosci Methods. 180(1):9-21.
